# Dataset on statistical reduction of highly water-soluble Cr (VI) into Cr (III) using RSM

**DOI:** 10.1016/j.dib.2018.12.054

**Published:** 2018-12-28

**Authors:** Anshu Yadav, Vinita Khandegar

**Affiliations:** University School of Chemical Technology, Guru Gobind Singh Indraprastha University, Dwarka, New Delhi 110078, India

**Keywords:** Cr (VI), Cr (III), EC, BBD, Fe, Al

## Abstract

With the excellent solubility, mobility, bioaccumulation and carcinogenesis, hexavalent chromium Cr (VI), widely exists in various industrial effluents such as chrome plating, metal finishing, pigments, and tanning. Cr (VI) is one of the toxic metal pollutants among all the heavy metals. Therefore, the purpose of this work was to convert highly water-soluble Cr (VI) into Cr (III) species using electrocoagulation (EC) process. The Box–Behnken design (BBD) as was applied to investigate the effects of major operating variables and optimization conditions. The predicted values of responses obtained using the model is agreed well with the experimental data. This work demonstrated that the Cr (VI) is entirely converted into Cr (III) in solid-phases in electrocoagulation process. It was also found that reduction increased with current density that suggesting that the reduction efficiency is closely related to the generation of floc.

**Specifications table**

**Table:** 

Subject area	Wastewater treatment
More specific subject area	Chemical engineering
Type of data	Table and Figure
How data was acquired	UV–vis Double Beam Spectrophotometer. (Hitachi U-2900, India)
Data format	Raw, analyzed
Experimental factor	Box–Behnken design matrix was used to investigate the effects of major operating variables using Al and Fe electrodes in batch EC process.
Experimental features	Removal of Cr (VI) by EC
Data source location	Guru Gobind Singh Indraprastha University, Dwarka New Delhi, India
Data accessibility	The data are available with this article

**Value of the data**

• Now day׳s the regulation of Cr (VI) in drinking water have spurred strong interests due its adverse effect of Cr(VI) on human as well ecosystem.
• The acquired data will be advantageous for the scientific community wanting to scale up and design an electrocoagulation process for removal of Cr (VI).
• Based on the dataset, electrocoagulation is considered as a promising treatment technology for wastewater such as electroplating, metal finishing, and tanning etc.
• The proposed design correlations may prove to be a useful tool in designing pilot and commercial plants for Cr (VI) removal.

## 1 Data

**Fig. 1:**
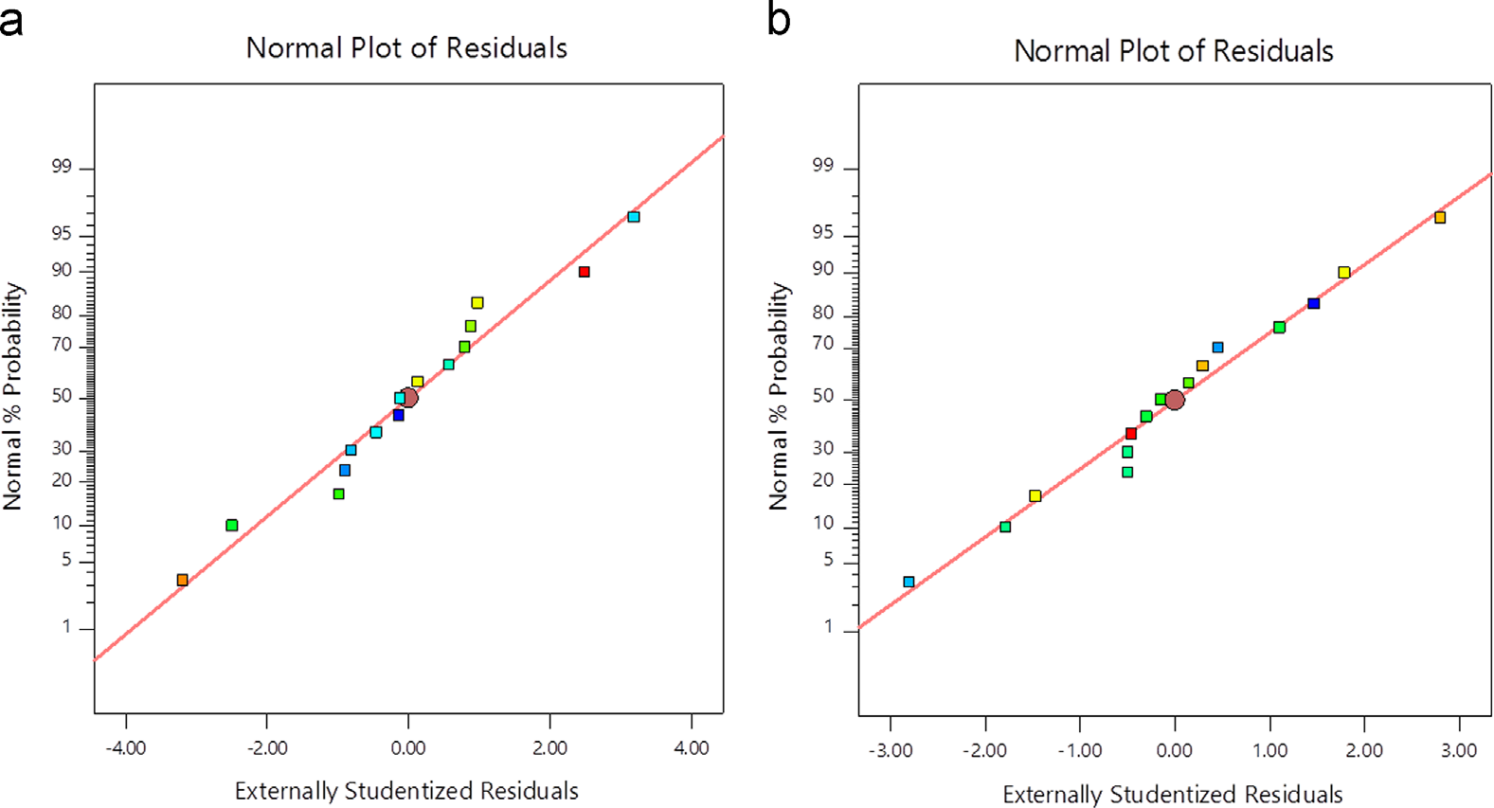
Normality probability plot (a) Al (b) Fe.

**Fig. 2:**
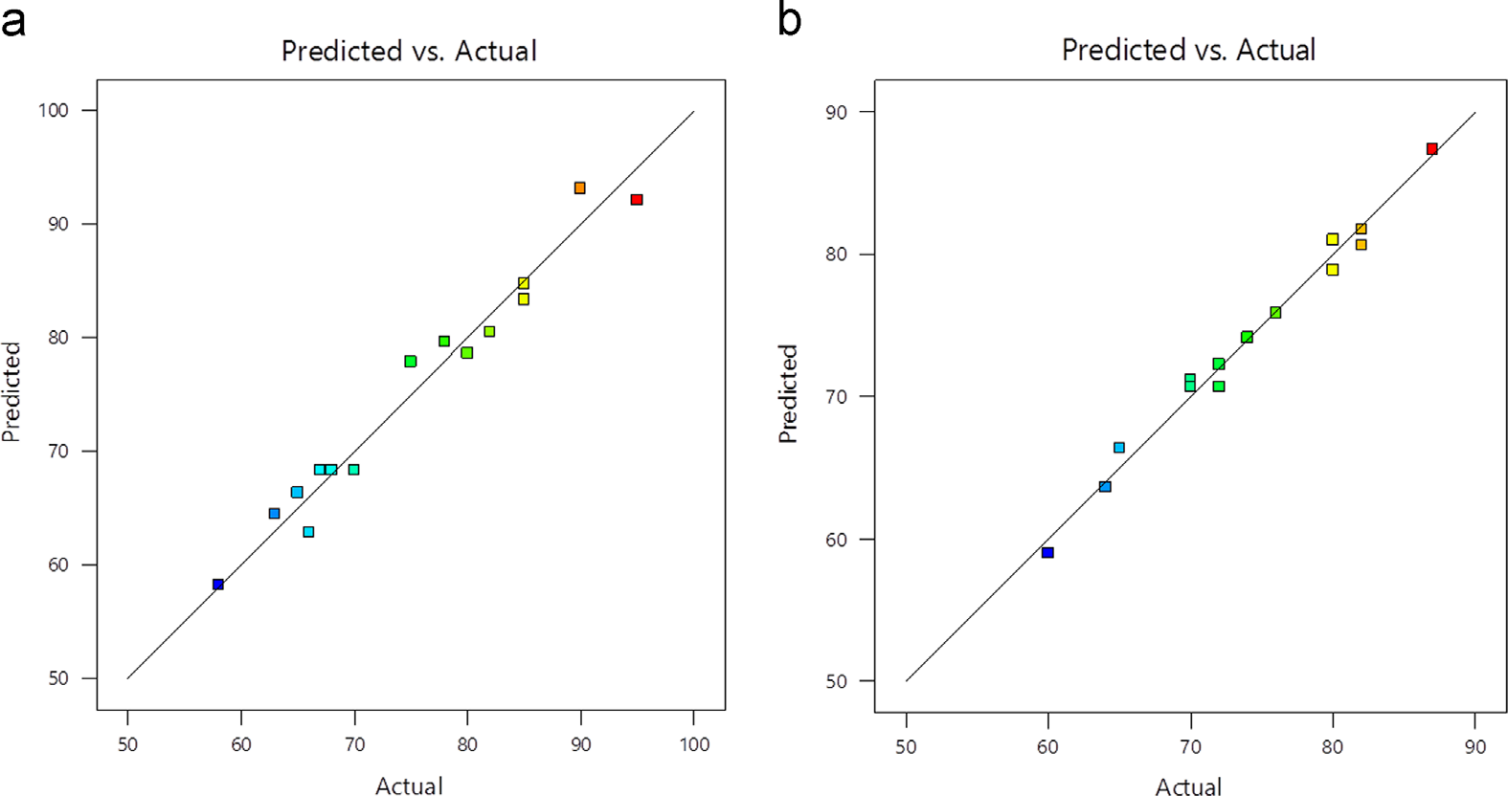
Predicted vs actual (a) Al (b) Fe.

**Fig. 3:**
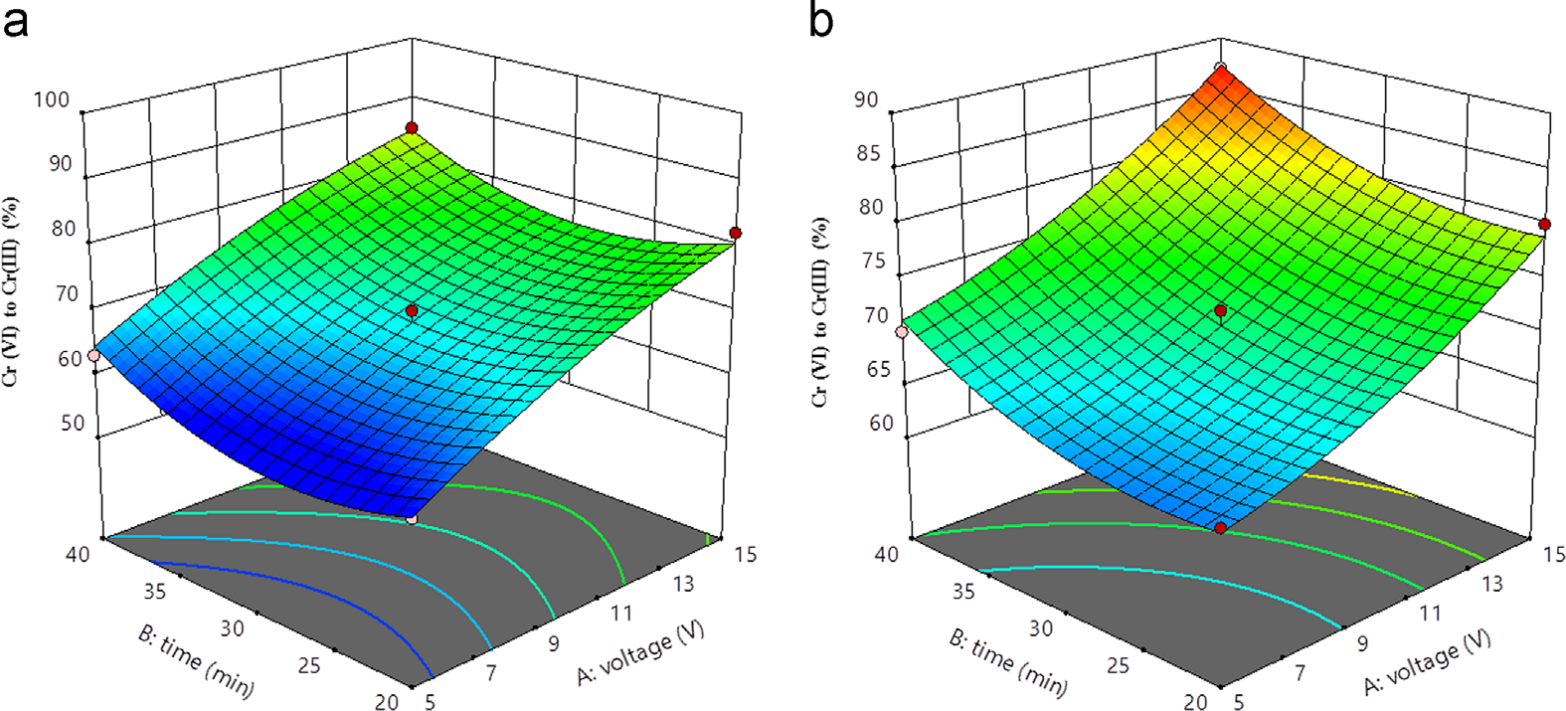
3D plot of time and voltage (a) Al (b) Fe.

**Fig. 4:**
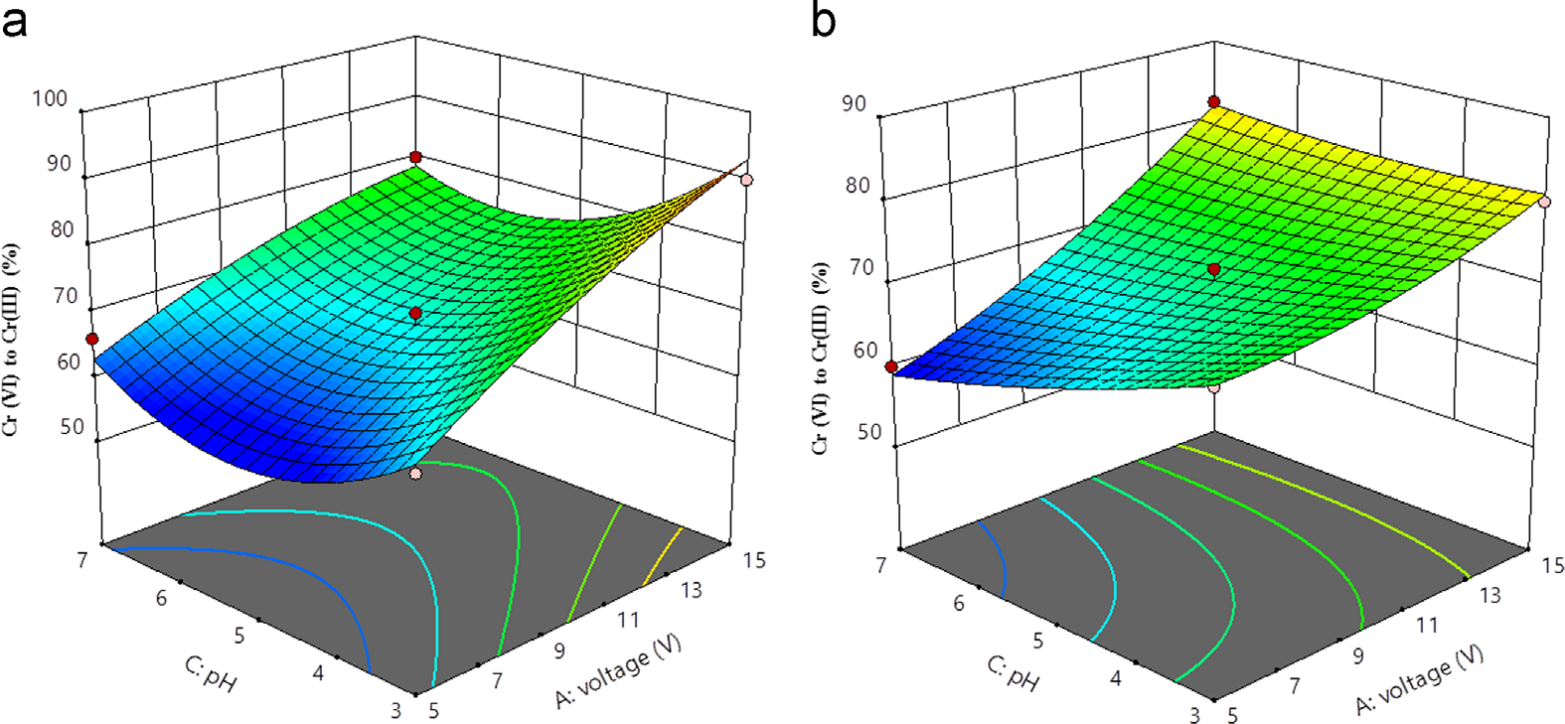
3D plot of pH and voltage (a) Al (b) Fe.

**Fig. 5:**
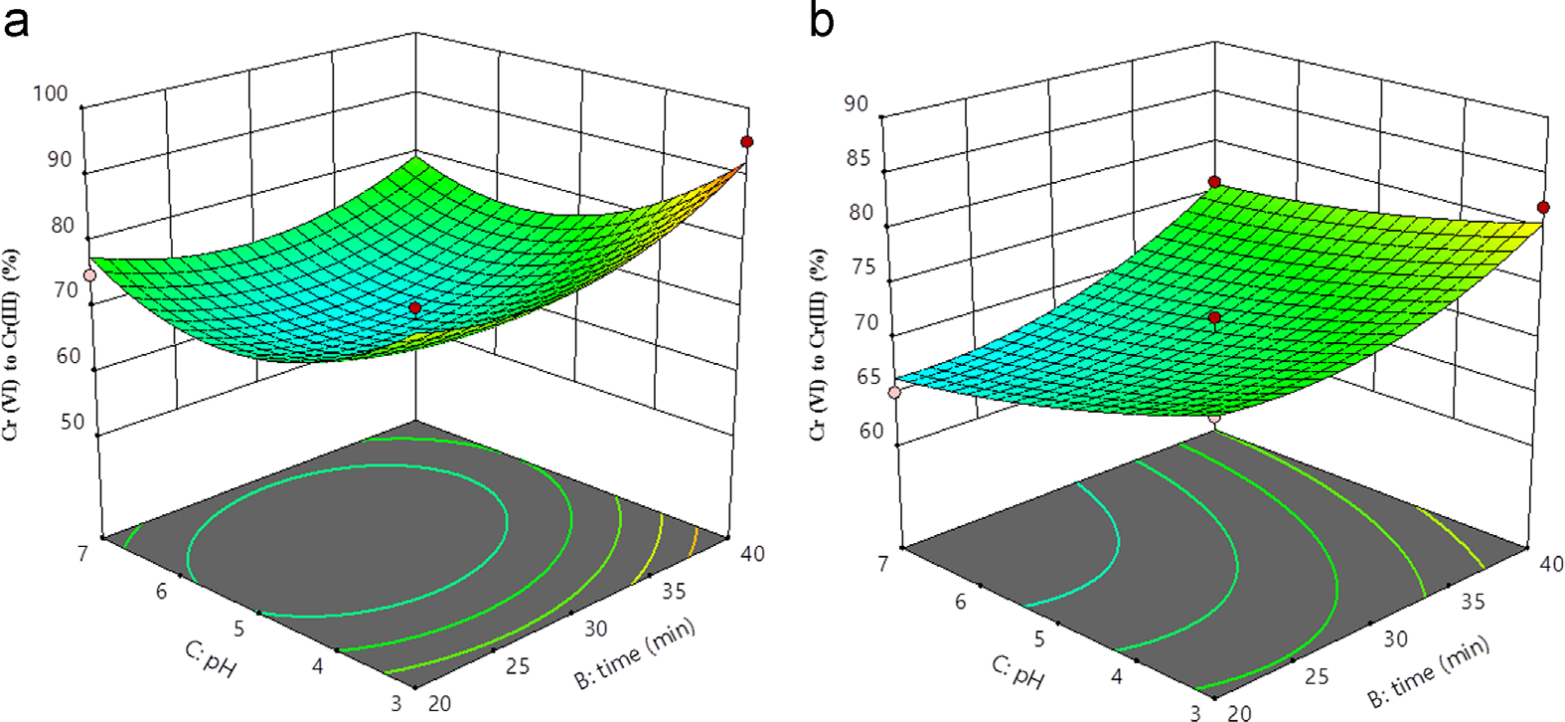
3D plot of pH and time (a) Al (b) Fe.

This dataset contains 3 Tables and 5 Figures that represent statistical optimization of electrocoagulation process for reduction of Cr (VI) to Cr (III) from synthetic wastewater in batch mode of operation using BBD. A total 15 number of batch experiments including three centre points were carried out in triplicates using statistically deigned experiments. The results are shown in Table 1, Table 2, Table 3a and 3b. The suitability of the selected model to provide adequate approximation of the real system is also confirmed by the diagnostic plots. Such plots include normal probability plots, residuals versus predicted and the predicted versus actual value plot (See Figs. 1 and 2). The 3D graphs were plotted to identify the optimized reaction conditions and to understand the individual effects of pH, voltage and time for efficient conversion of Cr (VI) to Cr (III) (See Fig. 3, Fig. 4, Fig. 5).

**Table 1:** Factor and levels of experiment through BBD.

**Factors**	**Levels**		
Voltage (V)	5	10	15
Time (min)	20	30	40
pH	3	5	7

**Table 2:** BBD matrix with experimental and predicted result.

**Run no.**	**Voltage (V)**	**Time (min)**	**pH**	**CRE (%)**			
				**Fe (Exp)**	**Fe (Pre)**	**Al (Exp)**	**Al (Pre)**
1	5	40	5	63.00	64.50	70.00	71.13
2	5	30	7	66.00	62.88	60.00	59.00
3	10	30	5	68.00	68.33	70.00	70.67
4	15	30	3	90.00	93.13	80.00	81.00
5	5	20	5	58.00	58.25	64.00	63.63
6	10	30	5	67.00	68.33	72.00	70.67
7	15	30	7	80.00	78.63	82.00	81.75
8	10	40	3	95.00	92.13	82.00	80.63
9	10	20	7	75.00	77.88	65.00	66.38
10	15	40	5	85.00	84.75	87.00	87.38
11	10	30	5	70.00	68.33	70.00	70.67
12	10	20	3	85.00	83.38	74.00	74.13
13	10	40	7	78.00	79.63	76.00	75.88
14	15	20	5	82.00	80.50	80.00	78.88
15	5	30	3	65.00	66.38	72.00	72.25

## 2 Experimental design, materials, and methods

### 2.1 Sample preparation

Stock solution (100 mg/L) of Cr (VI) was prepared by dissolving of potassium dichromate (Qualigens, India) in distilled water. Sodium hydroxide (Qualigens, India) and sulphuric acid (Qualigens, India) were used to adjust the pH of the solution. Potassium permanganate (Qualigens, India), sodium azide (Qualigens, India) and 1,5-diphenylcarbazide (Qualigens, India) were used for analysis of the chromium present in synthetic solution [1], [2].

### 2.2 Analytical methods

A 95 mL of Cr (VI) solution tested in a 100 mL volumetric flask. The pH of sample was maintained less than 2 by adding 2 drops of concentrated H_2_SO_4_ then 2 drops of phosphoric acid (H_3_PO_4_) were added. Then 2 mL of 1,5 Diphenylcarbazide (DPC) added to the solution and mixed thoroughly then leave for 5–10 min for full-color development. After full color development an appropriate amount of the solution (4 mL) was taken into 3 mm quartz cell and measured its absorbance at 540 nm using UV–vis Double Beam (Hitachi U-2900, India) spectrophotometer [3].

### 2.3 Experimental setup and procedure

The electrolytic cell consists of a glass beaker of 400 mL capacity. Aluminum and iron sheets were used as electrodes. The electrode distance between anode and cathode was maintained constant of 1.5 cm during electrolysis. A direct current was supplied by a DC power source (Science tech 4074, India, 0–5 A and 0–30 V). Agitation was provided to maintain uniform concentration inside the cell using (SPINOT 02, India). A stock solution Cr(VI) was prepared by dissolving an appropriate amount of potassium dichromate (Qualigens, India) in distilled water. All the experiments were carried out under potentiostatic conditions at room temperature. The pH of the solution was adjusted using either dilute HCl or NaOH. After each experiment the samples were collected and analyzed for Cr (VI) using 1,5-diphenylcarbazide (DPC) method.

The Cr (VI) to Cr (III) reduction percentage from synthetic solution was calculated using the following equation reported by [4], [5]:

(1) $$\mathrm{Cr}(\mathrm{VI})\;\mathrm{to}\;\mathrm{Cr}(\mathrm{III})\;(\%)=\left[\frac{C_{o}-C_{i}}{C_{o}}\right]\times 100$$

where *C_o_* and *C_i_* are initial and final concentrations of Cr (VI) in mg/L respectively. The reproducibility in the experimental results was found to be ±3%.

### 2.4 Statistical methods and data analysis

Box–Behnken design was established with the help of the Design Expert 11 software for statistical design of experiment and data analysis. The three significant process variables considered in this study were: Voltage (A), time (B) and pH (C) as shown in Table 1. The total number of experiments in this study was 15 including three center points were carried out in triplicates for the estimation of error. The observed and predicted results for each set of reaction parameters are given in Table 2. A quadratic polynomial equation using Design Expert software was fitted to the experimental data obtained according to the Box–Behnken design. Normality plot have been illustrated in Fig. 1 for aluminum (Al) and iron electrode (Fe) electrodes. Fig. 1 shows the normality assumption is clearly satisfied reduction of 95% which is close to result obtained by EC experiments given as straight line [6], [7]. The actual and the predicted results by EC process using aluminum and iron electrode is shown in Fig. 2. Actual values are the measured response data for a particular run, and the predicted values are evaluated from the model and generated by using the approximating function. It is seen in Fig. 2 that the data points lie close to the diagonal line and the developed model is adequate for the prediction of each response. ANOVA studies presented in Tables 3a and 3b. 3D plots (Fig. 3, Fig. 4, Fig. 5) suggested time and current as the dominant process parameters for reduction of Cr (VI) to Cr (III).

**Table 3a:** ANOVA analysis for Al electrode.

**Source**	**Sum of squares**	**df**	**Mean square**	***F*-value**	***p*-Value**	
**Model**	1605.32	9	178.37	16.39	0.0033	Significant
A-voltage	903.12	1	903.12	82.98	0.0003	
B-time	55.12	1	55.12	5.07	0.0742	
C-pH	162.00	1	162.00	14.89	0.0119	
AB	1.0000	1	1.0000	0.0919	0.7740	
AC	30.25	1	30.25	2.78	0.1564	
BC	12.25	1	12.25	1.13	0.3373	
A²	17.33	1	17.33	1.59	0.2626	
B²	125.64	1	125.64	11.54	0.0193	
C²	304.64	1	304.64	27.99	0.0032	
**Residual**	54.42	5	10.88			
Lack of Fit	49.75	3	16.58	7.11	0.1258	Not significant
Pure Error	4.67	2	2.33			
**Cor Total**	1659.73	14				
**Std. Dev.**	3.30	***R*^2^**				0.9672
**Mean**	75.13	**Adj *R*^2^**				0.9082
**C.V.%**	4.39	**Adeq. Precision**				12.9473

**Table 3b:** ANOVA analysis for Fe electrode.

**Source**	**Sum of squares**	**df**	**Mean square**	***F*-value**	***p*-Value**	
**Model**	792.18	9	88.02	38.55	0.0004	Significant
A-voltage	496.12	1	496.12	217.28	<0.0001	
B-time	128.00	1	128.00	56.06	0.0007	
C-pH	78.13	1	78.13	34.22	0.0021	
AB	0.2500	1	0.2500	0.1095	0.7541	
AC	49.00	1	49.00	21.46	0.0057	
BC	2.25	1	2.25	0.9854	0.3664	
A²	13.56	1	13.56	5.94	0.0588	
B²	26.26	1	26.26	11.50	0.0194	
C²	3.10	1	3.10	1.36	0.2963	
**Residual**	11.42	5	2.28			
Lack of Fit	8.75	3	2.92	2.19	0.3290	Not significant
Pure Error	2.67	2	1.33			
**Cor Total**	803.60	14				
**Std. Dev.**	1.51	***R*^2^**				0.9858
**Mean**	73.60	**Adjusted *R*^2^**				0.9602
**C.V.%**	2.05	**Adeq Precision**				22.9984
